# Factors associated with death in bedridden patients in China: A longitudinal study

**DOI:** 10.1371/journal.pone.0228423

**Published:** 2020-01-29

**Authors:** Jing Cao, Tiantian Wang, Zhen Li, Ge Liu, Ying Liu, Chen Zhu, Jing Jiao, Jiaqian Li, Fangfang Li, Hongpeng Liu, Huaping Liu, Baoyun Song, Jingfen Jin, Yilan Liu, Xianxiu Wen, Shouzhen Cheng, Xia Wan, Xinjuan Wu

**Affiliations:** 1 Department of Nursing, Chinese Academy of Medical Sciences, Peking Union Medical College, Peking Union Medical College Hospital, Beijing, China; 2 School of Nursing, Peking Union Medical College, Beijing, China; 3 Department of Nursing, Henan Provincial People’s Hospital, Zhengzhou, China; 4 Department of Nursing, The Second Affiliated Hospital, Zhejiang University School of Medicine, Hangzhou, China; 5 Department of Nursing, Wuhan Union Hospital, Wuhan, China; 6 Department of Nursing, Sichuan Provincial People's Hospital, Chengdu, China; 7 Department of Nursing, The First Affiliated Hospital, Sun Yat-sen University, Guangzhou, China; 8 Institute of Basic Medical Sciences, Chinese Academy of Medical Sciences and School of Basic Medicine, Peking Union Medical College, Beijing, China; Boston University, UNITED STATES

## Abstract

**Background:**

Immobility is common and associated with adverse outcomes in hospitalized patients, especially older people. However, the factors contributing to mortality in bedridden patients are not well known. This study aimed to estimate short-term mortality and analyze risk factors that affect the prognosis of bedridden patients.

**Methods:**

This was a multicenter study in China involving 23,738 patients admitted to 25 hospitals between November 2015 and June 2016. All-cause mortality was recorded for 90 days after enrollment regardless of whether death occurred before or after discharge. Socio-demographic and clinical information was obtained from an electronic database. Univariate and multivariate Cox regression analysis was used to identify factors associated with mortality.

**Results:**

In total, 23,738 hospitalized bedridden patients, there were 1,114 (4.7%) observed deaths. The overall mortality rate was therefore 4.7%. Of these, 318 (1.4%) died while hospitalized and 796 (3.4%) after discharge. The univariate Cox regression analysis showed that variables significantly associated with 90-day mortality included total time spent bedridden, urinary tract infection and pulmonary infection (*p*<0.05). The multivariate Cox regression analysis showed that the independent risk factors for death were age (adjusted hazard ratio [aHR] 1.006, 95% CI 1.000–1.011), and pulmonary infection (aHR 1.439, 95% CI 1.266–1.635). The hazard ratios for mortality were reduced with urinary tract infection and more time spent bedridden.

**Conclusions:**

The mortality after discharge was significantly higher than mortality in hospital. The factors affecting short-term mortality in bedridden patients included age, time spent bedridden, urinary tract infection and pulmonary infection. This suggests these factors may be potential predictors of mortality in bedridden patients. It is essential for medical staff to improve health education of patients and family members, pay more attention to follow up after discharge and meet care needs at home.

## Introduction

Immobility is a common phenomenon where patients are unable to move freely or physical movement is restricted [1]. Decreased activity and bed rest contribute to poor patient outcomes and carry substantial risk [2]. A study of 498 hospitalized older patients (≥ 70 years old) found that low mobility and bedrest were common and associated with adverse outcomes such as decline in ability to perform activities of daily living, death and institutionalization [3]. A prospective cohort study among 3,915 hospitalized older patients in 102 Italian internal medicine and geriatric hospital wards found that being bedridden was a risk factor for short-term mortality in non-oncological patients [4]. Another study [5] found that immobility was associated with risk of death in patients on invasive mechanical ventilation in an intensive care unit. Functional status is an important perioperative factor influencing patients’ outcomes. Scarborough [6] found that people who were functionally dependent generally had higher odds of mortality than those who were functionally independent. Immobile bariatric surgery patients had 4.59 fold greater odds of mortality, longer length of stay and readmissions and multiple complications than those who were mobile [7].

A prospective study found the top three complications in spinal cord injury patients were pressure ulcers (29.8%), pulmonary complications (23.4%) and urinary tract infections (17%) [8]. These immobility complications are also associated with longer length of stay in hospital [9] and higher mortality [10, 11]. We therefore assumed that immobility-related complications might contribute to death in bedridden patients.

It has been reported that the growing number of older people with chronic diseases is the driving force behind the high morbidity and mortality associated with immobility-related complications [12]. Despite a number of preventive strategies, chronic diseases and related complications remain prevalent and the high medical costs associated are a serious economic burden [13]. Various comorbidities including chronic renal failure, diabetes and congestive heart failure have also been found to be significantly associated with short-term (90-day) and long-term (90- to 180-day) mortality [14]. In China, the aging population has become an important social problem. The number of partially and completely disabled older adults has reached more than 40 million, and it is difficult for the care system to cope with large numbers of older people with functional disabilities or chronic diseases [15]. Immobility is more common among this group.

Immobility is therefore very common and related to poor patient outcomes, but the short-term mortality of bedridden hospitalized patients remains unclear, and the factors that contribute to mortality in those patients are also unknown. The objective of this study was therefore to estimate short term mortality and analyze associated risk factors that affect the prognosis of bedridden patients.

## Materials and methods

### Design

This was a longitudinal study in China. We carried out the study in 25 hospitals (six tertiary hospitals, 12 non-tertiary hospitals and seven community hospitals). The study participants were recruited from wards or departments with a large proportion of bedridden patients, including neurology, neurosurgery, geriatrics, orthopedics, thoracic surgery, and intensive care. Bedridden patients were defined as those for whom all basic physiological needs had been met in bed for at least 24 hours after admission. All the participants were adults (aged ≥ 18 years). The patients were observed and followed up for 90 days after enrollment in the study unless they died or gave up medical treatment. Patients who had more than one type of major immobility complication (pneumonia, pressure ulcer, deep vein thrombosis or urinary tract infection) at the beginning of the study were excluded.

### Data collection

Data were collected between November 2015 and June 2016. In each hospital, we appointed a coordinator to manage internal logistics. At least two registered nurses were selected as investigators and received standardized training on data collection using a Case Report Form (CRF). To ensure the quality of data, the project team members randomly selected 20% of cases to check the consistency of collected data with medical records. On-site examinations were used to identify problems quickly.

Socio-demographic information was obtained from the electronic database, included age, gender, education, smoking status, hospital type, physical performance, department, and Charlson comorbidity index score. Length of bedrest was defined as time spent bedridden before and during hospitalization. The International Classification of Diseases (ICD-10) codes were extracted from the database to identify diagnostic categories. Trained nurses assessed patients for pressure ulcers using the European and US National Pressure Ulcer Advisory Panels’ Pressure Ulcer Classification System. Pulmonary infection, deep venous thrombosis and urinary tract infection were determined from medical records.

All-cause mortality was recorded for 90 days after enrollment date regardless of whether death occurred before or after discharge. Survival time was defined as from time of enrollment to date of death. Date of death was collected from death certificates.

### Ethical considerations

The study was approved by the Ethical Committee of Peking Union Medical College Hospital. Data were collected by trained investigators using validated procedures and instruments. They provided verbal and written information about the study and obtained patients’ written consent to participate before the start of the study. If patients were unable to give written consent, their relatives were consulted about provision of consent. Participants were told that they could withdraw from the study at any time and that the care they received would not be affected. All patient information was kept confidential. This was an observational study and therefore did not cause any harm to patients.

### Data analysis

Categorical variables were expressed as counts and percentages. For normally-distributed data, continuous variables were expressed as mean and standard deviation (SD). After univariate Cox regression, clinically and statistically significant (*p* < 0.05) variables were included in the multivariate Cox proportional hazard ratio model, to identify risk factors associated with short-term mortality and estimate the adjusted hazard ratios (aHRs). All statistical analyses used SPSS version 22.0.

## Results

### Sample characteristics

From November 2015 to June 2016, 23,985 patients were recruited, of whom 247 were excluded because they had been bedridden for more than 100 days before enrollment. In total, therefore, 23,738 were included in the analysis. There were slightly more men than women (56.6% vs. 43.4%). The mean age was 57.0 ± 16.7 years (range 18–109 years), and 46.8% were over 60 years old. A total of 81.3% of patients were from tertiary hospitals. The prevalence of pneumonia, pressure ulcers, deep venous thrombosis, and urinary tract infection was 11.3%, 3.2%, 1.8% and 1.6%, and 7.4% were bedridden before their hospitalization. The median Charlson comorbidity index score was 3 and 25.1% scored more than 5. The median length of time confined to bed was 5 days, and median length of stay was 13 days (Table 1).

**Table 1:** Characteristics of bedridden hospitalized patients (N = 23,738).

Characteristics		Total N (%)	Survival N (%)	Dead N (%)
**Gender**	**males**	13436 (56.6)	12735 (56.3)	701 (62.9)
	**females**	10302 (43.4)	9889 (43.7)	413 (37.1)
**Age (year)**	**18–44**	5317 (22.4)	5340 (23.2)	77 (6.9)
	**45–59**	7306 (30.8)	7104 (31.4)	202 (18.1)
	**≥60**	11115 (46.8)	10280 (45.4)	835 (75.0)
**Education**	**Elementary school or below**	9408 (39.6)	8811 (38.9)	597 (53.6)
	**Junior/ senior high school**	10753 (45.3)	10339 (45.7)	414 (37.2)
	**Junior college or above**	3577 (15.1)	3474 (15.4)	103 (9.2)
**Smoking**	**Never smoking**	17357 (73.1)	16593 (73.3)	764 (68.6)
	**Quit smoking**	5205 (21.9)	4968 (22.0)	237 (21.3)
	**Smoking**	1176 (5.0)	1063 (4.7)	113 (10.1)
**Hospital type**	**Tertiary hospital**	19310 (81.3)	18519 (81.9)	791 (71.0)
	**Nontertiary hospital**	4119 (17.4)	3845 (17.0)	274 (24.6)
	**Community hospital**	309 (1.3)	260 (1.1)	49 (4.4)
**Type of immobility related complications**				
**Pressure ulcer**	**No**	22971 (96.8)	22000 (97.2)	971 (87.2)
	**Yes**	767 (3.2)	624 (2.8)	143 (12.8)
**Pulmonary infection**				
	**No**	21066 (88.7)	20439 (90.3)	627 (56.3)
	**Yes**	2672 (11.3)	2185 (9.7)	487 (43.7)
**Deep venous thrombosis**				
	**No**	23304 (98.2)	22227 (98.2)	1077 (96.7)
	**Yes**	434 (1.8)	397 (1.8)	37 (3.3)
**Urinary tract infection**				
	**No**	23359 (98.4)	22296 (98.6)	1063 (95.4)
	**Yes**	379 (1.6)	328 (1.4)	51 (4.6)
**Charlson comorbidity index score**				
	**0**	2939 (12.4)	2922 (12.9)	17 (1.5)
	**1~2**	6850 (28.9)	6737 (29.8)	113 (10.1)
	**3~4**	7989 (33.7)	7681 (34.0)	308 (27.6)
	**≥5**	5960 (25.1)	5284 (23.4)	676 (60.7)
**Bedridden time before enrollment (days)**				
	**0**	965 (4.1)	923 (4.1)	42 (3.8)
	**1~7**	21778 (91.7)	20776 (91.8)	1002 (89.9)
	**8~14**	532 (2.2)	510 (2.3)	22 (2.0)
	**≥15**	463 (2.0)	415 (1.8)	48 (4.3)
**Total bedridden time (days)**				
	**0~7**	15063 (63.5)	14610 (64.6)	453 (40.7)
	**8~14**	4613 (19.4)	4300 (19.0)	313 (28.1)
	**≥15**	4062 (17.1)	3714 (16.4)	348 (31.2)
**Length of stay (days)**				
	**≤7**	4344 (18.3)	4034 (17.8)	310 (27.8)
	**8~14**	9075 (38.2)	8749 (38.7)	326 (29.3)
	**≥15**	10216 (43.0)	9742 (43.1)	474 (42.5)
	**Missed data**	103 (0.4)	99 (0.4)	4 (0.4)

### Mortality

In total, 1,114 patients died during the 90-day follow-up period. This was an overall mortality rate of 4.7%, with 318 (1.4%) dying during hospitalization and 796 (3.4%) after discharge. The mean age of these patients was 69.1 ± 15.6 years, and 75% were over 60 years old. The top three most common diagnostic diseases in those who died were hypertension, pulmonary disease and heart failure. The mortality rate was higher in men than women (5.2% vs. 4.0%). The median Charlson comorbidity index score was 5, with 60.7% of patients scoring more than 5. The median time bedridden and length of stay were 9 and 12 days (Table 1).

### Risk factors associated with mortality

Table 2 shows the results of the univariate and multivariate Cox regression analysis. The univariate Cox regression analysis showed a number of variables that were significantly associated with short-term mortality (within 90 days), including total time spent bedridden, urinary tract infection and pulmonary infection. There were no significant differences by age, education, gender, Charlson comorbidity index score, and other complications (pressure ulcer and deep venous thrombosis).

**Table 2:** Cox proportional hazard model of survival in bedridden patients.

Characteristics	Univariate HR (95% CI)	*P*	Multivariate HR (95% CI)	*P*
**Age**	1.001 (0.997–1.004)	0.723	1.006 (1.000–1.011)	0.036*
**Gender**				
**male**	1.00		1.00	
**female**	0.981 (0.869–1.109)	0.762	0.947 (0.834–1.076)	0.405
**Education**				
**Elementary school or below**	1.00		1.00	
**Junior/ senior high school**	0.966 (0.852–1.095)	0.589	1.045 (0.910–1.201)	0.530
**Junior college or above**	1.102 (0.894–1.359)	0.362	1.205 (0.969–1.498)	0.094
**Total bedridden time**	0.991(0.988–0.995)	<0.001**	0.988 (0.984–0.992)	<0.001**
**Pressure ulcer**			——	
**No**	1.00			
**Yes**	1.119 (0.938–1.335)	0.212		
**Deep venous thrombosis**			——	
**No**	1.00			
**Yes**	1.038 (0.747–1.441)	0.825		
**Urinary tract infection**				
**No**	1.00		1.00	
**Yes**	0.569 (0.426–0.761)	<0.001**	0.613 (0.456–0.825)	0.001*
**Pulmonary infection**				
**No**	1.00		1.00	
**Yes**	1.230 (1.092–1.386)	0.001*	1.439 (1.266–1.635)	<0.001**
**Charlson comorbidity index score**				
**0**	1.00		1.00	
**1~2**	1.036 (0.622–1.726)	0.892	0.824 (0.490–1.385)	0.464
**3~4**	1.015 (0.622–1.654)	0.954	0.722 (0.428–1.218)	0.222
**≥5**	0.984 (0.608–1.593)	0.947	0.631 (0.370–1.077)	0.091

*p<0.05

** *p*<0.001

The multivariate Cox regression analysis found that age, total time spent bedridden, urinary tract infection and pulmonary infection were associated with death. Urinary tract infection and total time spent bedridden appeared to be protective. The adjusted hazard ratio (aHR) for death was 1.006 (95% CI 1.000–1.011) for those who were older, and 1.439 (95% CI 1.266–1.635) for those with pulmonary infections. The hazard ratios for mortality were reduced in patients with urinary tract infection and longer time spent bedridden (Table 2).

## Discussion

### Mortality

Our study found that overall short-time mortality among hospitalized bedridden patients was 4.7%, which is considerable. Short-term mortality (within three months) has many crucial implications in planning diagnosis, therapies and overall management [16]. A previous study [4] reported that three-month mortality after discharge was 5.4%, which was higher than in our study. However, the previous study included only 3,915 older people recruited from internal medicine and geriatric wards, of whom only 112 were bedridden [4]. The average age of those patients was older and comorbidities were more common. That study also focused on three-month mortality after discharge, which was different from our focus on mortality during and after hospitalization. That study also defined bedridden as the inability to walk or stand upright during the whole hospital stay, so the time spent bedridden was longer than in our study. It is possible that these factors were associated with higher mortality.

Our results showed that mortality after discharge was significantly greater than mortality in hospital. This may be because medical resources and care were better in hospital. It is also possible that patients’ condition may worsen after discharge, even though their acute symptoms were stable at discharge, and they would then need to receive timely treatment in their nursing home or rehabilitation facilities. Our hospitalized patients had a number of chronic non-infectious conditions (hypertension, pulmonary disease, heart failure, cerebral infarction). Patients with chronic conditions require care after discharge from a hospital. Clinical staff are crucial in transitional care management [17]. Pharmacist-led transitions of care services can reduce all causes of 30-day readmission rates in patients with congestive heart failure [18]. This may suggest that we should focus on transitional care management for patients after discharge to reduce the risk of death in this group.

### Associated risk factors in hospitalized bedridden patients

#### Age

Our results suggested that being older was an indicator of poor prognosis in bedridden patients. Aging and immobility are common contributors to functional decline especially in hospitalized older adults [2]. Older patients are considered more likely to develop medical complications during hospitalization, which may contribute to some adverse outcomes, such as high rates of functional disability, and increased lengths of stay [1, 3]. Two prospective studies found there was a linear relationship between decreasing life-space and increasing risk of mortality [19, 20]. These two studies recruited women or men with an average age of over 70 years living in the community. Increasing age is also associated with increasing incidence of pneumonia and likelihood of hospitalization and mortality [21, 22]. Overall, as the world population ages, more public health services will be required for older people.

#### Total time spent bedridden

One previous study found that being permanently bedridden was an independent predictor of 30-day mortality in geriatric patients[23]. Being bedridden for the whole stay in hospital was also a risk factor for three-month mortality after hospital discharge [4]. Our study, however, included any patients who stayed in bed for at least 24 hours after admission and we recorded total time spent bedridden before and during hospitalization. Our results showed time spent bedridden was a protective factor for 90-day mortality. Our patients may stay in bed for several reasons, including because of changes in their health, endoscopic operations, or because they have had surgical treatments. They had a number of chronic non-infectious conditions and they may be stable with a longer stay in bed.

We also found a positive relationship between time spent bedridden and length of stay, that is, more time spent bedridden was associated with longer length of stay. Length of stay is an important metric for both patients and healthcare providers. A previous study showed it protected against mortality in the US and Japan [24], where a longer length of stay after surgery in older hip fracture patients was significantly associated with lower risk of mortality. This might be because of different effects of chronic and acute diseases. In our study, approximately 19% of those who died had cardiocerebrovascular diseases. Patients with shorter hospital stays are more likely to have acute diseases that are often associated with death, such as heart disease and stroke [25]. However, chronic conditions require a sequence of complex treatment interventions, and this might prolong the time spent bedridden and length of stay, but not necessarily confer a high risk of mortality.

Overall, the study population, outcome indicators and study design are not uniform, and the results are not comparable. We therefore suggest that further research is needed on the relationship between time spent bedridden and mortality in bedridden patients.

#### Immobility-related complications

Pneumonia is still the leading cause of hospitalization and mortality worldwide [26], and 30-day mortality remains persistently high with this condition [12]. The multivariate analysis showed that pulmonary infection was associated with mortality, which is consistent with previous studies [12, 27, 28]. A cohort study, using the general population as controls, found that patients with pneumonia were almost 46 times as likely to die within 30 days as participants without pneumonia [28]. Another cohort study of 2465 hospitalized patients found that pneumonia was also associated with increased mortality beyond 30 days. People with pneumonia had increased cardiovascular mortality risk, although the reason for this was unclear. It is thought that pneumonia may have caused a chronic inflammatory response which could accelerate the process of cardiovascular disease [27]. Together with our findings, this suggests that healthcare providers should focus on patients with pneumonia to reduce their mortality.

Urinary tract infection is one of the most common in-hospital infections. [29] It can be further divided into simple or complicated, and the severity of infection varies significantly [30]. A retrospective study reported that urinary tract infection contributed to high 28-day mortality rate in hospitalized Chinese older patients [31]. Catheter‐associated urinary tract infection was also accompanied by an increase in mortality [32]. In our study, patients with urinary tract infection had a lower mortality rate, which was not consistent with precious conclusions. This may be because our study included a relatively small number of people with urinary tract infections, just 379 (1.6%). We also did not collect information about type and severity of infection, although these may be associated with different risks of mortality. Future studies should use a larger sample size to explore this relationship further.

We found no association between other complications of immobility (deep venous thrombosis and pressure ulcer) and mortality. Immobility during hospitalization has previously been found to increase the risk of venous thrombosis [33]. A review of 36 studies found evidence that mobilization brought physical benefits to hospitalized patients, including fewer deep venous thromboses and urinary tract infections, and lower incidence of pneumonia. However, these studies mainly focused on stroke and postoperative patients [34].

Over 60% of patients in our study were bedridden for no more than seven days. This may have been short enough to develop any adverse outcomes or the effects of low mobility. Further research is needed to explore the relationship between immobility-related complications and prognosis in bedridden patients.

#### Charlson comorbidity index score

The Charlson comorbidity index is widely used to predict short-term mortality and has been validated in various clinical populations [35]. In a previous study, a high Charlson comorbidity index score was an independent predictor of higher 1-year mortality in hospitalized heart failure patients [36]. Another study found a significant relationship between Charlson comorbidity index and 1-year mortality in Chinese older adults [37]. However, in our study, the Charlson comorbidity index score was not an independent predictor of survival. The distribution of scores was skewed, with 41.0% of the population assigned a score of 0 to 2, and only 25.3% having a score of 5 or over. The median score was 3, showing a moderate degree of comorbidity. More studies are needed to test the validity of Charlson comorbidity index for predicting mortality among bedridden patients. This finding warrants further investigation given the diversity of settings and diagnoses.

### Strengths and limitations

This study examined immobility-related complications and factors associated with mortality among bedridden patients, a group and subject that has not to our knowledge previously been explored. The multicenter investigation involving several hospitals and the large sample size means that the study can be considered representative of the broader population.

The study had several limitations, however. First, more than half the patients were bedridden for less than a week, which may have protected them from the adverse effects of immobility. Second, the medical diagnosis on death was not collected, so the major causes of death could not be analyzed. This information would be important for clinical work. However, the primary medical diagnoses on discharge were cardiovascular, cerebrovascular and lung conditions, so these may well have been the causes of death in our study. We also excluded patients who had spent over 100 days bedridden before enrollment, to reduce bias. More factors should be included in future studies to lay a foundation for better clinical work, including reasons for bedrest, and physical function.

## Conclusion

In total, 4.7% of the bedridden patients in this study died within 90 days of enrollment and the mortality after discharge was significantly higher than mortality during hospitalization. Factors associated with short-term mortality included age, time spent bedridden, urinary tract infection and pulmonary infection. This suggests these factors maybe be potential predictors of mortality in bedridden patients. More health services and policies may be needed to improve outcomes in this group. Medical staff should focus on bedridden patients to identify early signs of problems. Low mobility and bedrest are very common among older patients, who also need good medical care after discharge. It is essential for medical staff to improve health education of patients and family members, pay more attention to follow up after discharge and provide for care needs at home.
